# Role of USP13 in physiology and diseases

**DOI:** 10.3389/fmolb.2022.977122

**Published:** 2022-09-14

**Authors:** Qian Wang, Zhenzhen Sun, Weiwei Xia, Le Sun, Yang Du, Yue Zhang, Zhanjun Jia

**Affiliations:** ^1^ Nanjing Key Laboratory of Pediatrics, Children’s Hospital of Nanjing Medical University, Nanjing, China; ^2^ Jiangsu Key Laboratory of Pediatrics, Nanjing Medical University, Nanjing, China; ^3^ Department of Nephrology, Children’s Hospital of Nanjing Medical University, Nanjing, China

**Keywords:** USP13, physiology, regulation, diseases, cancers

## Abstract

Ubiquitin specific protease (USP)-13 is a deubiquitinase that removes ubiquitin from substrates to prevent protein degradation by the proteasome. Currently, the roles of USP13 in physiology and pathology have been reported. In physiology, USP13 is highly associated with cell cycle regulation, DNA damage repair, myoblast differentiation, quality control of the endoplasmic reticulum, and autophagy. In pathology, it has been reported that USP13 is important in the pathogenesis of infection, inflammation, idiopathic pulmonary fibrosis (IPF), neurodegenerative diseases, and cancers. This mini-review summarizes the most recent advances in USP13 studies involving its pathophysiological roles in different conditions and provides new insights into the prevention and treatment of relevant diseases, as well as further research on USP13.

## Introduction

Ubiquitination is a dynamic posttranslational modification that mostly leads to protein degradation by the 26S proteasome while simultaneously, regulating multiple cellular processes including the cell cycle, cell death and signal transduction (Critchley et al., 2018; Dang et al., 2021; Sahu and Glickman, 2021; Roberts et al., 2022). Similarly, removal of ubiquitin from conjunct substrates by deubiquitination also affects the abovementioned biological processes. Ubiquitination is sequentially catalyzed by ubiquitin (Ub)-activating enzymes (E1), Ub-conjugating enzymes (E2), and Ub ligases (E3), while deubiquitination is mediated by deubiquitinating enzymes (DUBs). Nearly 100 DUBs have been identified and they can be divided into seven subclasses: Ub c-terminal hydrolase (UCH), Ub specific protease (USP), ovarian tumor protease (OTU), Josephin (JOS), JAB1/MPN/MOV34 metalloenzyme (JAMM), motif-interacting with Ub (MIU)-containing novel DUB (MINDY), and Zinc finger with UFM1-specific peptidase domain protein (ZUFSP) (Darling et al., 2017; Kwasna et al., 2018; Suresh et al., 2020a). Additionally, monocyte chemotactic protein-induced protein 1 (MCPIP1), an important participant in inflammation, also possessed a deubiquitinating activity and contained a novel DUB domain differing from the above seven DUBs (Liang et al., 2010). Besides, JAMMs are Zn^2+^ metalloproteases, and the others are all cysteine proteases. DUBs recognize their substrates by either binding to proteins or directly interacting with specific Ub-chain types.

USPs are the largest family of DUBs and are composed of a core catalytic domain and other functional domains such as the ubiquitin-associated (UBA) domain, ubiquitin interacting motif (UIM), and zinc finger (ZNF) domain (Bonnet et al., 2008; Komander et al., 2009; Ye et al., 2009). Although the catalytic domains of USPs differ in size and sequence, a structure simulating palm, thumb, and finger is highly conserved. The palm and thumb subdomains jointly form the active site and the finger subdomain assists Ub in entering the catalytic center (Reyes-Turcu and Wilkinson, 2009).

USP13, early known as Isopeptidase T (ISOT-3), was discovered by Timms et al., (1998). In structure, compared with other members in USP family, USP13 and USP5 (also known as ISOT-1) have the highest similarity, 67.3% identity in nucleotide and 54.8% identify in amino acid (Timms et al., 1998). USP5 is a well-featured member in USP family which harbors a ZNF domain, two UBA domains, and a catalytic domain (Timms et al., 1998; Reyes-Turcu et al., 2008). Functionally, unlike the efficient deubiquitinating activity of USP5, USP13 displayed a very low catalytic activity which might be explained by the defect in the ZNF domain of USP13 (Zhang et al., 2011). Notably, the tandem UBA domains of USP13 could bind to Ub and catalyze the hydrolysis of the polyUb chains (Lys-63/48/27) (Zhang et al., 2011; Sun et al., 2017; Xie et al., 2020a). Unfortunately, the crystal structure of human USP13 has not been analyzed, which limits the development of USP13 agonists or antagonists. Though possessing weak catalytic activity, recent studies have described the involvement of USP13 in various pathophysiological processes by preventing the proteolysis of its substrates. In this mini-review, we summarized the documented substrates of USP13 and its role in various physiological and pathological states, as well as prospects for future research.

## Ubiquitin specific protease-13 regulates the cell cycle

The eukaryotic cell cycle is the process of DNA genome duplication and its division into daughter cells, consisting the interphase and the Mitotic (M) phase. The interphase is responsible for DNA duplication and synthesis of RNA, proteins, and ribosomes, prepared well for mitosis. The M phase, is a continuous process going through prophase, metaphase, anaphase, and telophase. The cell cycle is a complicated network involving various cyclins, cyclin-dependent kinases (CDKs) and other regulators (Teixeira and Reed, 2013; Matthews et al., 2022). In this precise procedure, many proteins are regulated by multiple post-translational modifications including phosphorylation, SUMOylation, and ubiquitination et al. (Eifler and Vertegaal, 2015; Zou and Lin, 2021; Krasinska and Fisher, 2022). In this part, we focus on the ubiquitination or deubiquitination of some regulators mediated by USP13 in the cell cycle regulation and may provide some insights for future research.

Aurora kinase B (AuroraB), a vital mitotic protein kinase, forms a chromosomal passenger complex (CPC) with the inner centromere protein and survivin. AuroraB functions by phosphorylating some proteins involved in centromeric functions and mitosis (Zeitlin et al., 2001; Goto et al., 2002; Goto et al., 2003). The anaphase-promoting complex/cyclosome (APC/C) is a E3 ligase involved in mitotic progression with two key adaptors, the cell division cycle 20 (CDC20) and the cadherin 1 (CDH1) (Fang et al., 1998; Hall et al., 2004). It has been reported that AuroraB is a substrate of APC/C and especially AuroraB forms a complex with CDH1 during mitosis (Stewart and Fang, 2005). USP13 was reported to regulate the stability of AuroraB to affect the progression of the cell cycle. Overexpression of USP13 could induce AuroraB accumulation, which increased the phosphorylation of histone H3, leading to G2 or prophase arrest; however, USP13 knockdown reduced the level of AuroraB, resulting in stuck in G1 phase (Esposito et al., 2020). Shortly after this finding, the interaction of USP13 with CDH1 was also confirmed by the same team (Esposito and Gutierrez, 2022). The USP13-APC/C^CDH1^-AuroraB axis forms a new regulatory model in the cell cycle progression. Therefore, maintaining the proper level and activity of USP13 helps to stabilize cell cycle progression by targeting APC/C^CDH1^-AuroraB. Cohesin is a ring-like multiple protein complex holding the sister chromatids together throughout G2 phase until the start of mitosis, and phosphorylation of Cohesin allows its removal from chromosome arms and the subsequent sister chromosome separation (Haarhuis et al., 2014). Mass spectrometry analysis revealed that USP13 could interact with Cohesin, and USP13 functioned as both ubiquitinase and deubiquitinase for Cohesin subunits. USP13 deficiency led to total loss of ubiquitin of Cohesin subunits rather than increased ubiquitination. Besides, the ubiquitination of Cohesin is essential for its disassociation from chromatin (He et al., 2020). Therefore, USP13 is required for Cohesin ubiquitination and its release from chromatin during mitosis. This broadens our knowledge of USP13 in addition to its deubiquitinating activity; however, how USP13 acts as a “ubiquitinase” remains to be solved.

**FIGURE 1:**
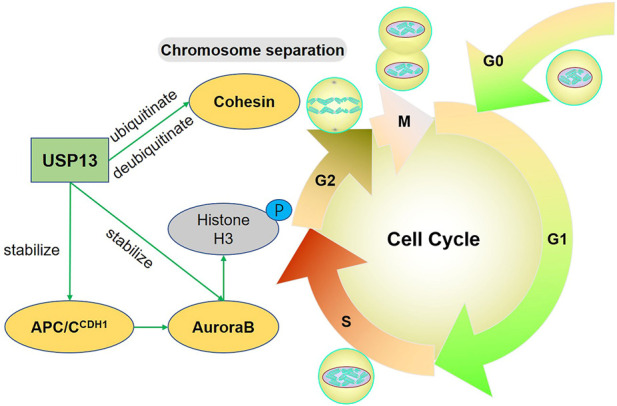
USP13 regulates the cell cycle. USP13 stabilizes APC/C^CDH1^-AuroraB-axis to facilitate chromatin remodeling and condensation. Particularly, AuroraB functions by phosphorylating multiple proteins involved in the mitosis, such as Histone H3. Cohesin is ubiquitinated by USP13 to help sister chromosome separation during mitosis and simultaneously USP13 can also deubiquitinate and stabilize Cohesin. Green arrow indicates promotion.

Conclusively, USP13 directly stabilizes AuroraB or indirectly stabilizes its upstream modulator CDH1 to maintain AuroraB level, ensuring the successful entry of cells into M phase. Cohesin ubiquitin by USP13 is also required for sister chromatin separation during mitosis (Figure 1).

## Ubiquitin specific protease-13 regulates DNA damage response

**FIGURE 2:**
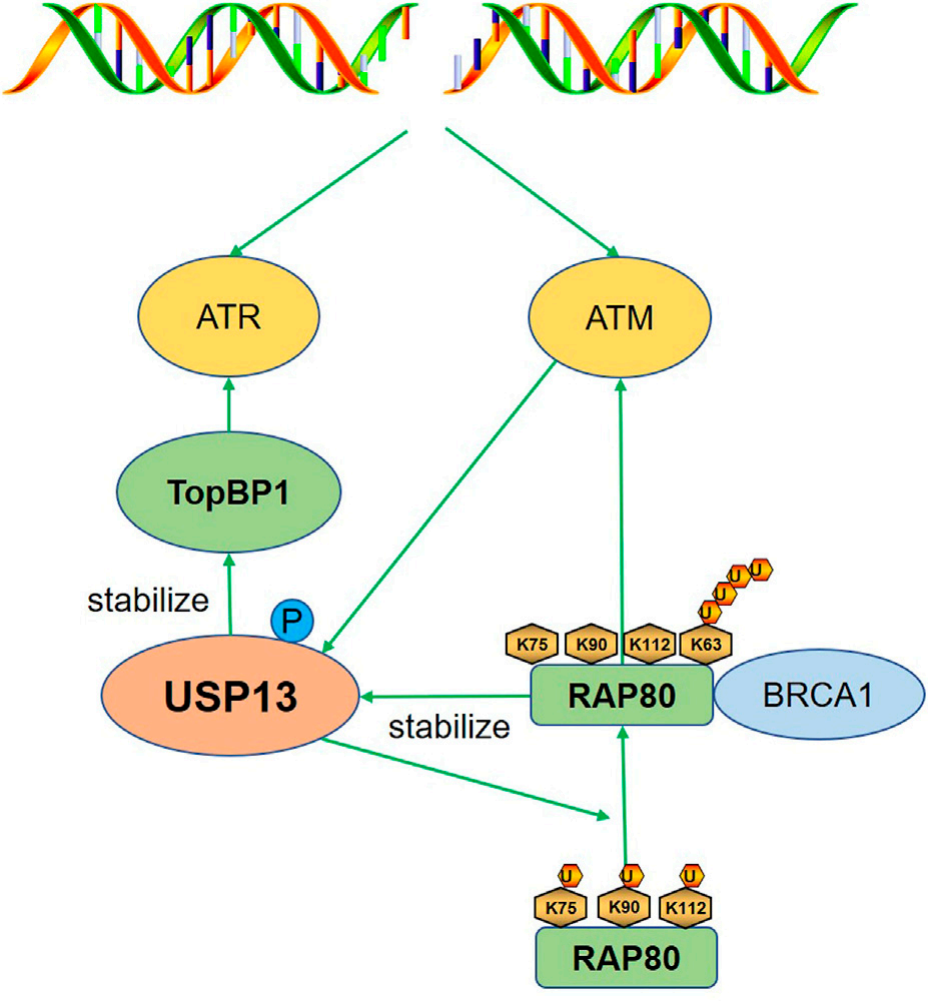
USP13 regulates DDR. After DNA damage, USP13 is phosphorylated and activated by ATM to deubiquitinate RAP80 (K75, K90, and K112), which promoted the binding of RAP80 with the K63-linked polyubiquitin chain and the subsequent recruitment of BRCA1, triggering proper DDR. TopBP1 is also stabilized to activate ATR in response to replication stress. Green arrow indicates promotion.

DNA damage response (DDR) is a complicated regulatory system that senses DNA lesions or replication stress and protects genomic stability and integrity with different responses such as cell cycle delay, DNA repair, senescence, and apoptosis et al. (Jackson and Bartek, 2009; Ciccia and Elledge, 2010). The ataxia-telangiectasia mutated (ATM)-checkpoint kinase 2 (Chk2) and the ataxia-telangiectasia mutated and Rrad3-related (ATR)-checkpoint kinase 1 (Chk1) are two key pathways involved in DDR (Petsalaki and Zachos, 2020). Here we discussed the function of USP13 in DDR by stabilizing several effectors including RAP80 and TopBP1 (Figure 2).

Receptor-associated protein 80 (RAP80) is a breast cancer susceptibility gene 1 (BRCA1)-interacting protein that could recruit BRCA1, the principle function of which is to facilitate homologous recombination (HR), to sites of DNA breaks after DNA damage (Kim et al., 2007). A recent study suggested that DNA damage could induce the phosphorylation of USP13 by the ATM and then activated USP13 deubiquitinated RAP80 (K75, K90, and K112). Notably, the deubiquitination of the three key sites facilitated the binding of RAP80 with the K63-linked polyubiquitin chain; therefore, RAP80 could recruit BRCA1 to double-strand breaks (DSBs) and trigger a proper DNA damage response (DDR) (Li et al., 2017). This suggests deubiquitination is important for the binding of RAP80 to the K63 polyubiquitin chain, which may be a result of the exposure of the binding site. Still, the underlying mechanism is not clear and warrants further investigation. Conversely, RAP80 could stabilize USP13 to maintain efficient DSB repair, providing a safer microenvironment for esophageal cancer cell growth (Yang et al., 2018). The positive feedback between USP13 and RAP80 is of great significance for genomic stability in normal cells, whereas it promotes cancer cells proliferation. Therefore, inhibit USP13 or RAP80 may increase the efficacy of anti-tumor drugs, and the consequent suppression of DDR should be concerned.

Topoisomerase IIβ-binding protein (TopBP1) is a replication repair protein recruited to stalled replication forks to activate ATR (Makiniemi et al., 2001; Kumagai et al., 2006). USP13 was recently identified as a deubiquitinase for TopBP1 and was important for replication checkpoint activation by stabilizing TopBP1 (Kim et al., 2021).

Therefore, USP13 plays a considerable role in maintaining genomic stability *via* regulation of RAP80 and TopBP1. Considering the importance of DDR in cancer therapy, targeting USP13 may synergize with anti-tumor drugs. However, there have been some literatures illustrating controversial roles of USP13 in different kinds of cancers, which is discussed in later sections. Therefore, more investigations are required to draw a comprehensive regulating spectrum by USP13.

## Ubiquitin specific protease-13 regulates myoblast differentiation

The development of skeletal muscle usually goes through two periods: the embryonic stage characterized by the proliferation of myogenic cells and the post-natal muscle grows largely by differentiation (Braun and Gautel, 2011). Recently, it was reported USP13 performed differently in lamb and fetus muscles with low expression in fetus while gradually increased expression in lamb muscles (Zhang et al., 2022a). Besides, silencing USP13 inhibited the differentiation of goat primary myoblasts (GPMs), suggesting USP13 may positively regulate myoblast differentiation. However, the mechanism in which USP13 regulates myoblast differentiation remains to be investigated. Considering the importance of skeletal muscle for energy metabolism and locomotion, it is meaningful to evaluate the role of USP13 in skeletal muscle development and USP13 may act as a potential target in some congenital or acquired muscle diseases.

## Ubiquitin specific protease-13 regulates endoplasmic reticulum quality control

The endoplasmic reticulum (ER) is an organelle responsible for the processing of membrane and secretory proteins. Only properly folded proteins can be destinated to where it functions while misfolded proteins are eliminated by the ER-associated degradation (ERAD) system (Smith et al., 2011). Different E3 ubiquitin ligases are the centers of ERAD pathways, such as gp78, also known as autocrine motility factor receptor (AMFR) (Fang et al., 2001). The Bag6 complex composed of Bag6, Ubl4A, and Trc35 is recognized as a chaperone molecule of ubiquitin ligases including gp78. The core component Bag6 promotes ERAD by maintaining the soluble state of ERAD substrates owing to its holdase activity (Wang et al., 2011). Notably, the opposite gp78 and USP13 both facilitated ERAD. The polyubiquitination of the Bag6 cofactor Ubl4A by gp78 could lead to inactivation of Bag6, while USP13 could simultaneously remove the polyubiquitination of Ubl4A to preserve the holdase activity of Bag6 (Liu et al., 2014). This suggests the deubiquitination by USP13 sometimes does not affect the target protein level.

Ubiquitin fusion degradation 1 (UFD1) is an adaptor protein that plays a part in ERAD (Ye et al., 2001). Generally, UFD1, together with the valosin containing protein (VCP) and the NPL4 homolog, ubiquitin recognition factor (NPLOC4) to form a ternary complex, which could bind with the ubiquitinated proteins and export them for degradation (Nowis et al., 2006). UFD1 was reported to function as a scaffold for the USP13-Skp2 (the F-box adaptor of the E3 ubiquitin ligase SCF^Skp2^) interaction, maintaining Skp2 stability. Under prolonged ER stress, the downregulation of UFD1 reduced the deubiquitination of Skp2 by USP13, leading to the degradation of Skp2 and the resulting cyclin-dependent kinase inhibitor p27 (a major substrate of SCF^Skp2^) accumulation (Chen et al., 2011). The increased level of p27 led to G1 delay and therefore accelerated the clearance of misfolded proteins by ERAD. Recently, it was reported spautin-1, an unspecific inhibitor of USP13, significantly suppressed unfolded protein response (UPR) and reduced cell viability during glucose starvation. However, this reduction of UPR was independent of USP13 inhibition because silencing USP13 did not cause obvious effects on UPR (Kunimasa et al., 2022). This phenomenon may be explained by off-target effects and the limitation of unspecific inhibitors urges the development of novel selective USP13 inhibitors. These data suggest USP13 may promote ERAD *via* modulation of Ubl4A and Skp2; however, whether other proteins involved in ERAD could be regulated by USP13 remains to be investigated.

## Ubiquitin specific protease-13 regulates autophagy

**FIGURE 3:**
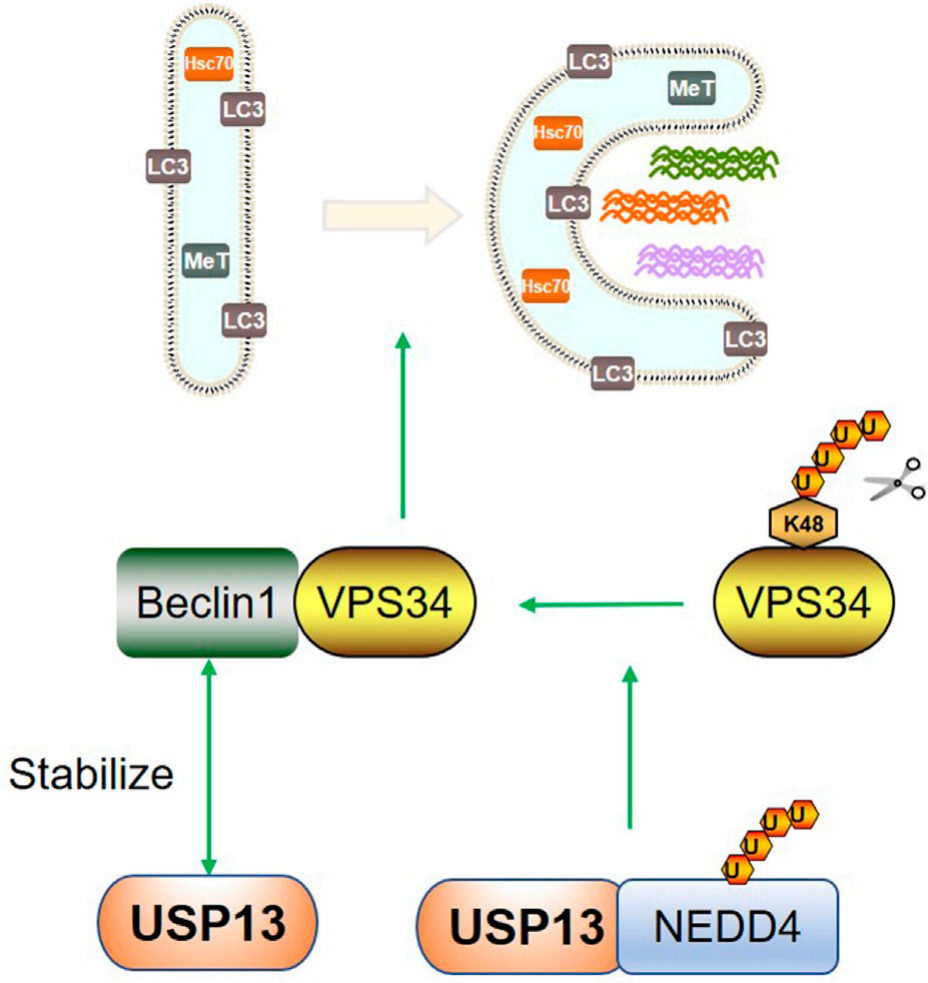
USP13 regulates autophagy. USP13 and Beclin1 can interact with each other. USP13 can coordinate with auto-ubiquitinated NEDD4 to remove K48 linked polyubiquitin chain of VPS34 to promote autophagy. Green arrow indicates promotion.

In addition to ER quality control, autophagy also plays an indispensable role in cellular hemostasis regulation. Autophagy is an adaptive process eliminating misfolded or aggregated proteins, old or damaged organelles, and invasive pathogens (Dikic and Elazar, 2018). Autophagy is well controlled by two kinases complexes, two ubiquitin-like conjugation systems, and a shuttling protein. The multimolecular PtdIns 3-kinase class III (PtdIns3K/VPS34)-serine/threonine kinase p150 (VPS15)-Beclin1 (Atg6) complex is one of the two kinase systems, which is required for autophagy induction. The two ubiquitin-like conjugation systems, the Atg12-Atg5-Atg16 and LC3-phosphatidylethanolamine (-PE), are mainly responsible for the elongation and expansion of autophagic membrane (Dewaele et al., 2010). There are various posttranslational modifications in Beclin1 and VPS34, including ubiquitination. Intriguingly, a previous study revealed that USP13 and Beclin1 could interact with each other. Pharmacological inhibition of USP13 with spautin-1 reduced the levels of VPS34 complexes especially Beclin1 while overexpression of USP13 inhibited the ubiquitination degradation of Beclin1; on the other hand, the interaction of Beclin1 with USP13 significantly enhanced the DUB activity of USP13 (Liu et al., 2011). Although the mechanism for the regulation of Beclin1 on USP13 DUB activity was not illustrated, other substrates such as RAP80 also showed a similar contribution to USP13 activity. We may speculate USP13 can also be modulated by multiple posttranslational modifications including ubiquitination. Besides, the crosstalk between USP13 and other proteins may change its conformation, which probably affects its DUB activity. Except for Beclin1, VPS34 was recently recognized as a substrate of USP13. USP13, together with the autoubiquitinated HECT ubiquitin E3 ligase NEDD4-1, forms a deubiquitination complex, which can remove the K48-linked polyubiquitin chain of VPS34 to stabilize VPS34 and thus advance autophagy (Xie et al., 2020b). The above results indicate USP13 promotes autophagy by targeting the VPS34 complex. In addition, the phenotypic switch of microtubule-associated protein light chain 3 (LC3) (LC3-Ⅰ to LC3-Ⅱ) is a feature of the autophagic membrane. Recently, cleaved Bag6 induced by caspase three activation was reported to interact with LC3-Ⅰ and pro-LC3 under ER stress conditions such as gp78 overexpression and USP13 downregulation, leading to autophagy inhibition and apoptosis activation (Chu et al., 2020). Mechanistically, under ER stress, the ubiquitination of thioredoxin (TXN), a common substrate of gp78 and USP13, promoted caspase three activation and the consequent apoptosis. These results demonstrate USP13 could not only cooperate with other factors such as NEDD4-1 but also directly interact with Beclin1 to maintain normal autophagy by stabilizing the VPS34 complex under unstressed state, whereas downregulated USP13 triggered by various stimuli could lead to autophagy depression due to some indirect effects. Also, autophagy is a double-edged sword in which proper autophagy promotes cell survival while overactivated autophagy causes cell injury and death. Therefore, targeting USP13 to regulate autophagy remains to be investigated. Figure 3 summarized the regulation of USP13 in autophagy.

## Ubiquitin specific protease-13 in disease conditions

In addition to its role in cell cycle regulation, DDR, myoblast differentiation, ER quality control and autophagy, USP13 is also involved in the pathogenesis and development of various pathological conditions, including infection, inflammation, fibrosis, neurodegenerative diseases and cancers.

## Ubiquitin specific protease-13 in infection

Interferons (IFNs) are prominent executors defending against viruses and signal transducer and activator of transcription 1/2 (STAT1/2) are important participants in the signaling cascade of IFNs (Aaronson and Horvath, 2002). The phosphorylated STAT1 homodimer or the heterodimer formed by STAT1 and STAT2 can respectively form the gamma interferon activated sequence (GAF) or the interferon-stimulated gamma factor-3 (ISGF3), which could translocate to the nucleus and initiate IFN-gene transcription (Tolomeo et al., 2022). RNA interference screening identified USP13 as a regulator of IFN-α activity toward dengue virus serotype 2 (DEN-2) (Yeh et al., 2013). *In vitro*, knockdown of USP13 weakened the resistance of cells to DEN-2 probably by reducing the stability of STAT1. However, whether USP13 can enhance the antiviral response *in vivo* has not been documented. Besides, except STAT1, whether other anti-virus signaling pathways such as RIG-I/MAVS pathway could also be regulated by USP13 deserves to be investigated.

Besides STAT1, stimulator interferon genes (STING) plays core roles in the regulation of innate immune response to DNA virus infection (Ishikawa and Barber, 2008; Cai et al., 2014). In general, cytosolic DNA is sensed by the cyclic-GMP-AMP (cGAMP) synthase (cGAS) which catalyzes the synthesis of cGAMP. Then, activated STING by cGAMP recruits TBK1, leading to IRF3 phosphorylation and its translocation to nucleus to induce IFNα and other cytokines production (Cai et al., 2014). In contrast to the positive regulation on the STAT1-mediated antiviral response, USP13 was found to remove the K27-linked polyubiquitin chain of STING, which did not affect the protein level of STING while restraining TANK binding kinase 1 (TBK1) recruitment to STING and therefore suppressing DNA virus-triggered signaling (Sun et al., 2017). This negative modulation may be explained by keeping the basal immune response under physiological control and preventing extreme immune responses and inflammation under viral infection. Compared to WT mice, USP13 deletion rendered mice more resistance to herpes simplex virus type 1 (HSV-1) infection, possibly a result of an enhanced STING-mediated antiviral response resulting from USP13 deficiency (Sun et al., 2017). Besides, the regulation of USP13 on STING has some similarity with that of RAP80, both not influencing the protein levels of substrates while affecting the binding of substrates with other factors. This indicates USP13 mostly protects proteins from proteasomal degradation *via* deubiquitination, whereas this deubiquitination sometimes functions as a regulator of substrates depending on the role of mono- or poly-ubiquitination modification.

Additionally, severe acute respiratory syndrome coronavirus 2 (SARS-CoV-2) is threatening people’s health worldwide by damaging the immune system. New research has shown that SARS-CoV-2 nonstructural protein 13 (NSP13) can hijack host USP13 to stabilize itself. In addition, the interaction of NSP13 and TBK1 hindered the recruitment of TBK1 to mitochondrial antiviral signaling protein (MAVS), and therefore suppressed IFNs production (Guo et al., 2021). Although knockdown or inhibition of USP13 rescued the reduced IFN-β induced by NSP13 and inhibited virus replication, further studies are warranted to assess the efficacy of USP13 inhibitors in SARS-CoV-2 infection. We expect that USP13 inhibitors exhibit satisfactory efficacy in animal models and USP13 serves as a potential target for the treatment of SARS-COV-2.

**FIGURE 4:**
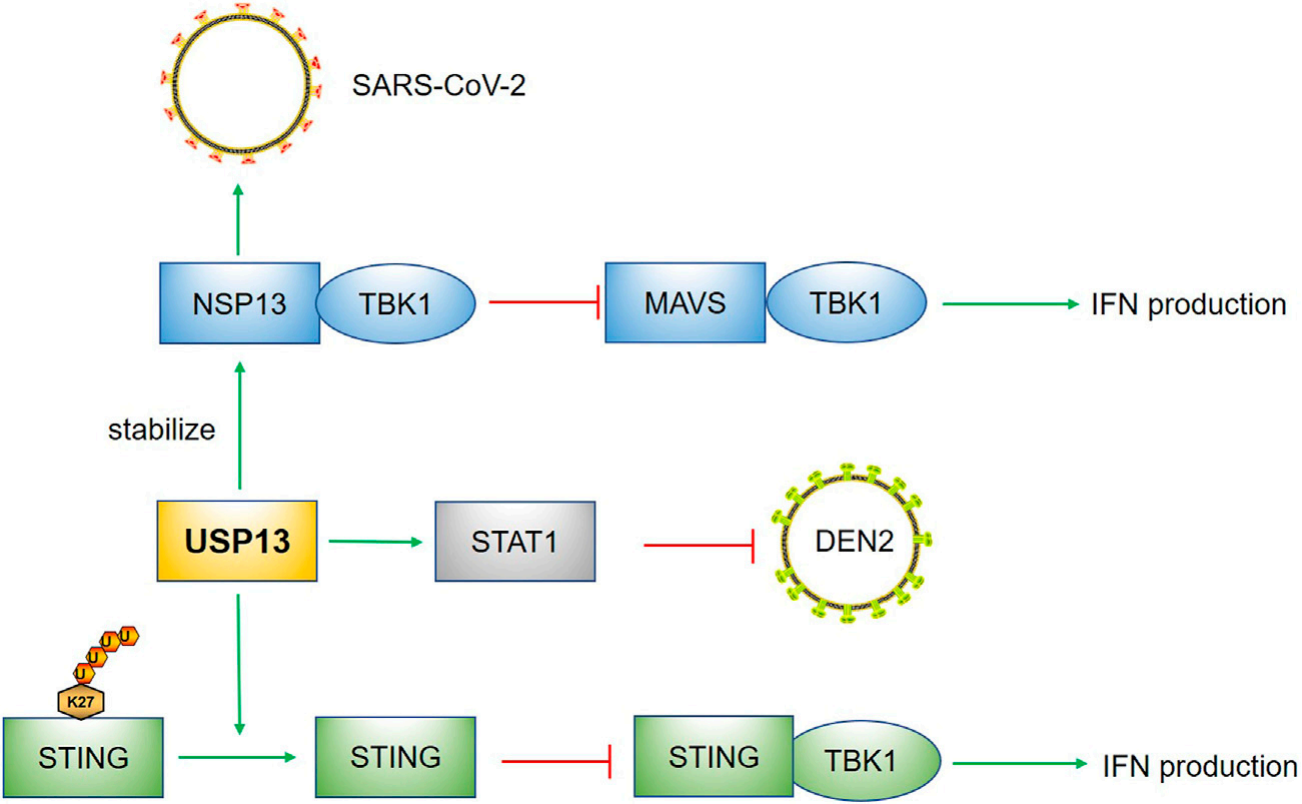
USP13 in infection. USP13 stabilized STAT1 to suppress DEN2. The removal of K27 linked polyubiquitin chain of STING by USP13 hindered the recruitment of TBK1 to STING, leading to reduction of IFN production. The NSP13 of SARS-COV-2 could hijack USP13 to stabilize itself and the binding of NSP13 with TBK1 prevented MAVS-TBK1 signaling pathway. Green arrow indicates promotion; Red capped line represents inhibition.

In summary, these data suggest USP13 performs differently by deubiquitinating multiple substrates in virus infection, which is summarized in Figure 4. Due to the diversity of viruses, revealing the roles of USP13 in other viruses is also significant. Besides the abovementioned STAT1, STING, and MAVS-mediated anti-virus signaling pathways, other pathways may also be regulated by USP13. In addition, infection does not only include virus, but also involve bacteria, fungus, parasites and other microbes. Therefore, whether USP13 has regulatory effects on other microbial infection needs to be evaluated.

## Ubiquitin specific protease-13 in inflammation

Inflammation is a kind of defensive response against various stimuli, involving multiple signaling pathways such as NF-κB, PI3K-AKT, and MAPK et al. There have some reports documented the regulation of inflammation by USP13. The single immunoglobin interleukin-1 (IL-1)-related receptor (Sigirr), also named IL-1R8, functions as an anti-inflammatory receptor by negatively regulating IL-1 and Toll-like receptor (TLR) signaling (Qin et al., 2005). In lipopolysaccharide (LPS)-induced mice, USP13 and Sigirr were consistently downregulated (Li et al., 2019; Yu et al., 2021). USP13^−/−^ (USP13 gene knockout) mice displayed exacerbated lung inflammation, while overexpression of Sigirr reversed the enhanced lung injury observed in USP13-deficient mice. This may be partly explained by the deubiquitination and stabilization of Sigirr by USP13 (Li et al., 2019). Consistently, in another study, USP13 deletion increased LPS-induced proinflammatory cytokine production, which was related to NF-κB and P38 activation (Wang et al., 2021). Though USP13 also deubiquitinated interleukin-1 receptor-associated kinase (IRAK)4, a common upstream of NF-κB and P38, USP13 deficiency did not reduce the protein level of IRAK4, while it increased IRAK4 phosphorylation and thereby downstream signaling. Therefore, the inhibitory effects of USP13 on inflammation may be independent of its regulation of IRAK4, and there may be other regulatory mechanisms. The lipid phosphatase PTEN (phosphatase and tensin homolog) is a negative regulator of serine/threonine kinase AKT, which plays a crucial role in inflammation and oxidative stress (Worby and Dixon, 2014; Liu et al., 2020). PTEN has been recognized as a substrate of USP13 (Zhang et al., 2013). Recently, USP13 exhibited protective effects on osteoarthritis, which was partly dependent on PTEN-mediated AKT deactivation (Huang et al., 2021). These data suggest USP13 suppresses the inflammatory response by targeting Sigirr and PTEN. However, in another study, USP13 was involved in NLRP3 inflammasome activation. NLRP3 inflammasome signaling is another proinflammatory pathway which amplifies the inflammatory response by promoting the maturation and excretion of interleukin-1β (IL-1β) and IL-18 (Schroder and Tschopp, 2010). Upstream of the NLRP3 inflammasome, the plasma membrane channel P2X7 receptor (P2X7R) and the adaptor protein Paxillin cooperated to activate the NLRP3 inflammasome (Wang et al., 2020). Notably, in this process, USP13 was required for paxillin-mediated deubiquitination and oligomerization of NLRP3 (Wang et al., 2020). Therefore, due to the diversity and complexity of inflammatory factors and pathways, the role of USP13 in inflammation remains unclear and needs more research.

## Ubiquitin specific protease-13 in idiopathic pulmonary fibrosis

**FIGURE 5:**
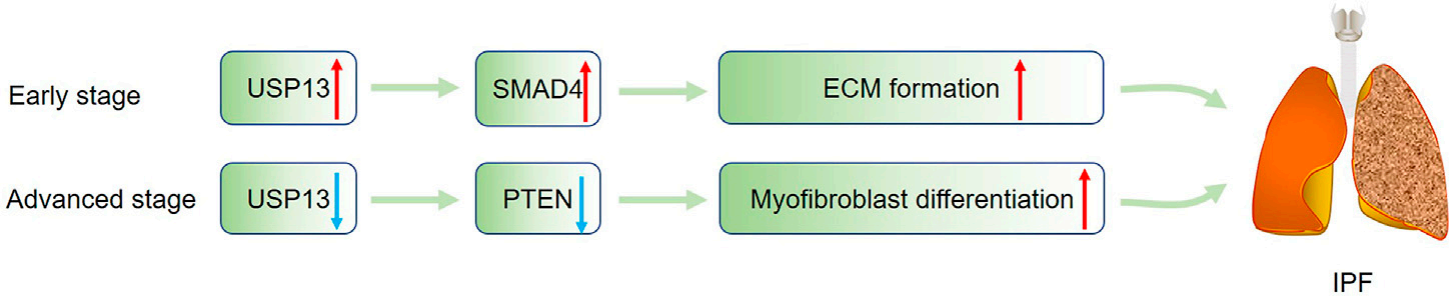
USP13 in IPF. During the progression of IPF, USP13 is upregulated to stabilize SMAD4 to promote ECM formation in the early stage, and in the late stage, hypoxia-induced USP13 reduction could lead to PTEN downregulation, facilitating myofibroblast differentiation. Green arrow indicates upregulation; Blue arrow represents downregulation.

Idiopathic pulmonary fibrosis (IPF) is a progressive interstitial lung disease characterized by fibroblastic foci formation (Selman et al., 2001). The cause of IPF remains unknown, and no effective pharmacological therapies are available for patients (Suri et al., 2021). Therefore, illustrating the pathogenesis of IPF may help to develop new treatment for IPF. A previous study showed USP13 expression was significantly decreased in lung tissues and fibroblasts from IPF patients, and knockdown of USP13 in lung fibroblasts enhanced the proliferative, migratory and invasive capacity of fibroblasts, suggesting USP13 insufficiency may contribute to IPF progression (Geng et al., 2015). Further investigation revealed the contribution of USP13 deficiency to IPF may be related to the downregulation of PTEN, which has been reported to promote myofibroblast differentiation (White et al., 2006). Strangely, differing from the downregulation of USP13 in IPF lungs, USP13 was markedly increased in fibroblasts isolated from mice challenged with bleomycin (BLM) and in TGF-β1-treated lung fibroblasts, accompanied by the increased expression of FN and collagen (Liao et al., 2020). The author claimed that the upregulated USP13 might promote extracellular matrix (ECM) formation by deubiquitinating and stabilizing SMAD4, one could form a trimeric complex with SMAD2/3 to initiate relevant gene transcription (Moustakas et al., 2001). How to explain the discrepancy of USP13 expression in IPF patients and BLM-treated mice? We speculate during the early stage of IPF, USP13 is upregulated to accelerate ECM by stabilizing SMAD4; however, in the advanced stage, hypoxia can induce USP13 downregulation and the resultant PTEN reduction, leading to phenotypic switch of fibroblasts (Figure 5). Additionally, it has been claimed that IPF is associated with immune cells-mediated injury and inflammation, whether USP13 regulates immune and inflammatory responses in IPF deserves to be determined (van Geffen et al., 2021). Besides lung fibrosis, liver and kidney fibrosis are also common clinical diseases, and the function of USP13 in these diseases remains to be explored. Furthermore, except for the abovementioned TGF-β/SMAD signaling, Wnt/β-catenin, MAPK, and Notch pathways all participate in the pathogenesis of fibrosis, and whether USP13 regulates these pathways needs more research.

## Ubiquitin specific protease-13 in neurodegenerative diseases

Neurodegenerative diseases are characterized by progressive loss of structure and function of neurons (Tesco and Lomoio, 2022). Currently, although large quantities of studies regarding neurodegenerative diseases have been published, the mechanism is still obscure. More importantly, these diseases are difficult to treat and bring huge burden for the society and families. Protein aggregation are the pathological features of several neurodegenerative diseases, amyloid-β for Alzheimer’s disease (AD) and α-synuclein for Parkinson’s disease (PD) for example (Vaquer-Alicea and Diamond, 2019; Sengupta and Kayed, 2022). These aggregates can be removed by the ubiquitin-proteasome degradation system, in which process USP13 can play a part.

Alzheimer’s disease is the most common neurodegenerative disease, pathologically manifested as amyloid-β(Aβ) deposition and the accumulation of toxic hyperphosphorylated tau (p-tau) (Duyckaerts et al., 2009). Liu et al., (2019a) reported USP13 expression was markedly increased in postmortem AD brains and that knockdown of USP13 could significantly reduce amyloid plaque and tau accumulation in transgenic animal AD models of human amyloid precursor protein (APP) (TgAPP) or P301L tau mutations (rTg4510). Directly, the interaction of p-tau and ubiquitin was enhanced in mice injected with shRNA USP13, leading to increased elimination of p-tau by the proteasome pathway (Liu et al., 2019a). In addition, USP13 knockdown was found to slightly increase autophagic activity and 20S proteasome activity, both contributing to p-tau clearance. Since previous studies have demonstrated USP13 potentiated autophagy, more evidence is warranted to support this subtle increase in autophagy induced by USP13 knockdown (Liu et al., 2011; Xie et al., 2020b). Additionally, parkin, an E3-ubiquitin ligase involved in autophagy, was reported to reduce intracellular Aβ levels and extracellular Aβ plaque formation in AD models by promoting autophagy (Khandelwal et al., 2011). Whether USP13 regulates other autophagy-relevant proteins such as parkin to promote Aβ and tau clearance in AD remains to be evaluated.

Parkinson’s disease, the second neurodegenerative disorder, is manifested as α-synuclein aggregate-induced neuron loss and Lewy body formation (Bloem et al., 2021). Similar to AD, increased levels of USP13 were also detected in the brains of PD patients, which is considered a protection of α-synuclein to facilitate the PD process (Liu et al., 2019b). Furthermore, USP13 knockdown markedly reversed the motor performance of PD mice induced by lentiviral α-synuclein transfer, functioning as indirect evidence for the regulation of USP13 on α-synuclein. Also, pharmacological inhibition of USP13 could lower the level of α-synuclein and improve motor and behavior defects (Liu et al., 2021; Liu et al., 2022). Previous studies also demonstrated Parkin ubiquitination could promote the autophagic clearance of α-synuclein (Lonskaya et al., 2013; Madsen et al., 2021); however, the increased α-synuclein ubiquitination resulting from USP13 knockdown was independent of Parkin, though USP13 reduction also increased Parkin ubiquitination and proteasome activity (Liu et al., 2019b). Therefore, though USP13 could regulate autophagy, USP13-mediated ubiquitin-proteasome degradation and autophagy-induced clearance may be two independent pathways in PD.

Collectively, these findings suggest developing novel, specific USP13 inhibitors may be a strategy to prevent and treat neurodegenerative diseases such as AD and PD. However, mounting evidence have demonstrated impaired autophagy is associated with degenerative disorders and autophagy induction can ameliorate experimental symptoms, and notably some small molecular modulators have entered clinical trials (Suresh et al., 2020b; Park et al., 2020). As we discussed in the former section, USP13 could positively regulate autophagy. In this aspect, inhibiting USP13 may lead to autophagy reduction to some extend and the efficacy of USP13 inhibition in models of neurodegenerative diseases should be carefully assessed.

## Ubiquitin specific protease-13 in cancers

Although numerous studies have demonstrated the involvement of USP13 in various tumors by deubiquitinating multiple substrates, the exact function of USP13 in tumors remains controversial.

Some research has revealed that USP13 contributes to the pathogenesis and development of tumors. Myeloid cell leukemia sequence 1 (MCL-1), an anti-apoptotic protein belonging to the BCL-2 family, is also known as an oncogenic protein (Hata et al., 2015; Senichkin et al., 2019). Analysis of the copy number of The Cancer Genome Atlas (TCGA) tumor samples showed that USP13 and MCL-1 were upregulated in many types of tumors, especially in lung adenocarcinoma, lung squamous cell carcinoma, ovarian cancer, and cervical cancer (Zhang et al., 2018; Wu et al., 2019; Morgan et al., 2021). *In vitro* assays revealed the interaction of USP13 and MCL-1 and USP13 could deubiquitinate and stabilize MCL-1 (Zhang et al., 2018; Morgan et al., 2021). Besides, USP13 depletion dramatically inhibited cancer cell proliferation and suppressed tumor growth in xenograft models while reintroduction of MCL-1 partly restored cell proliferation and increased the tumor volume, reinforcing the regulation of MCL-1 by USP13 (Zhang et al., 2018; Wu et al., 2019; Morgan et al., 2021). Recently, in a novel ovarian cancer mouse model, overexpression of USP13 with deletion of PTEN and Trp53 (two important tumor suppressors) significantly enhanced the tumorigenic and metastatic properties of ovarian cancer (Kwon et al., 2022). Additionally, increased USP13 expression was observed in hepatic hepatocellular carcinoma (HCC) and high level of USP13 was correlated with larger tumor size, advanced tumor-node-metastasis (TNM) stage, and lower overall survival (Gao et al., 2020; Huang et al., 2020). Except MCL-1, c-Myc is also an oncogene and is highly expressed in many cancers (Lin et al., 2012). In glioblastoma multiforme (GBM), USP13 was found to deubiquitinate and stabilize c-Myc, which was required for maintaining glioma stem cell (GSC) self-renewal and tumorigenic potential (Fang et al., 2017). Thus, disrupting USP13 to reduce c-Myc protein levels may impair GSC maintenance and effectively restrain GBM tumor growth. In addition, USP13 was upregulated in melanoma and pharmacological inhibition with spautin-1 or genetic knockdown of USP13 both inhibited melanoma cell growth (Zhao et al., 2011; Guo et al., 2020). In particular, microphthalmia-associated transcription factor (MITF) is regulated by USP13 at the posttranslational level (Zhao et al., 2011). Notably, only an intermediate level of MITF promotes proliferation, while a high MITF level leads to cell differentiation and a low MITF level predisposes cells to cell cycle arrest and apoptosis (Gray-Schopfer et al., 2007). Therefore, targeting USP13 to reduce MITF levels could suppress melanoma by inducing cell apoptosis. Toll-like receptor 4 (TLR4), an essential sensor involved in carcinogenesis, is also a substrate of USP13, and TLR4/myeloid differentiation factor 88 (MyD88)/NF-κB pathway activation facilitates the proliferation, migration and invasion of HCC cells (Chen et al., 2008; Gao et al., 2020). Additionally, USP13 was upregulated in gastric cancer, and this upregulation may promote advanced tumor stage by stabilizing Snail, an inducer of epithelial–mesenchymal transition (EMT) and metastasis (Zhang et al., 2022b). In addition to the abovementioned oncogenic proteins, USP13 also accelerates tumor progression by regulating cancer metabolism. ATP citrate lyase (ACLY) and oxoglutarate dehydrogenase (OGDH), two key enzymes responsible for TCA cycle metabolism, were identified as USP13 substrates (Han et al., 2016). In ovarian cancer (OVCA), amplified USP13 promoted core metabolic pathways by stabilizing ACLY and OGDH, providing enough ATP, reducing equivalents, and precursors for lipid biosynthesis for OVCA cell growth (Han et al., 2016). Besides ACLY and OGDH, fatty acid synthase (FASN) was recently identified as a novel substrate of USP13 in small cell lung cancer (SCLC). USP13-mediated FASN stability promoted SCLC stemness and lipogenesis, accelerating SCLC tumor growth (Wang et al., 2022). Considering the importance of metabolic pathways in cancer progression, application of selective inhibitors of above-mentioned key enzymes may coordinate to treat cancer with anti-tumor drugs. Additionally, a specific USP13 variant (c.1483G>A) was identified in some papillary thyroid carcinoma (PTC) patients. Also, *in vitro* results indicated USP13 knockdown decreased thyroid cancer cell proliferation while USP13 c.1483G>A variant increased colony formation. These suggest USP13 may be a contributor to PTC and this mutation enhanced its carcinogenicity (Maria et al., 2022). Collectively, these data indicate the enhanced proliferation and invasion of cancers including lung cancer, OVCA, HCC, PTC, and GBM are positively related to increased USP13 levels or USP13 mutation and genetic or pharmacological inhibition of USP13 exhibits notable suppression of tumor growth and invasion. Developing USP13 inhibitors may provide novel insights into cancer therapy.

At the same time, some studies have illustrated the inhibitory effects of USP13 on carcinogenesis. The tumor suppressor PTEN has been recognized as a substrate of USP13 (Garcia-Cao et al., 2012; Zhang et al., 2013). Downregulated USP13 was correlated with PTEN levels in human breast carcinomas and overexpression of USP13 exhibited inhibitory effects on the PTEN-positive breast cancer cell line MDA-MB-231 but not the PTEN-null cell line BT549 (Zhang et al., 2013). Besides breast cancer, reduced USP13 expression was also detected in human bladder cancer (BC), oral squamous carcinoma (OSCC), human colorectal cancers, and testicular embryonal carcinoma (Xiang et al., 2015; Cheung et al., 2016; Man et al., 2019; Qu et al., 2019). Knockdown of USP13 significantly increased the proliferation, migration, and invasion of BC cells, which was largely rescued by PTEN reintroduction (Man et al., 2019). Mechanistically, USP13 was negatively regulated by the NF-κB/miR-130b/301b axis, and in contrast, USP13-induced loss of PTEN promoted persistent AKT and NF-κB activation, forming a regulatory cycle (Taniguchi and Karin, 2018). In addition, reduced PTEN-mediated AKT activation also allows cancer cells to survive and grow by stimulating glycolysis (Elstrom et al., 2004; Qu et al., 2019). Overexpression of USP13 remarkably decreased the protein levels of glucose transporter-1 (GLUT1) and hexokinase-2 (HK2) and therefore reduced glucose uptake and lactate production, which are essential energy providers for tumor cells (Qu et al., 2019). These data suggest the inhibition of USP13 in the abovementioned tumors probably mainly depends on PETN, as no other tumor suppressors have been identified as direct substrates of USP13.

**FIGURE 6:**
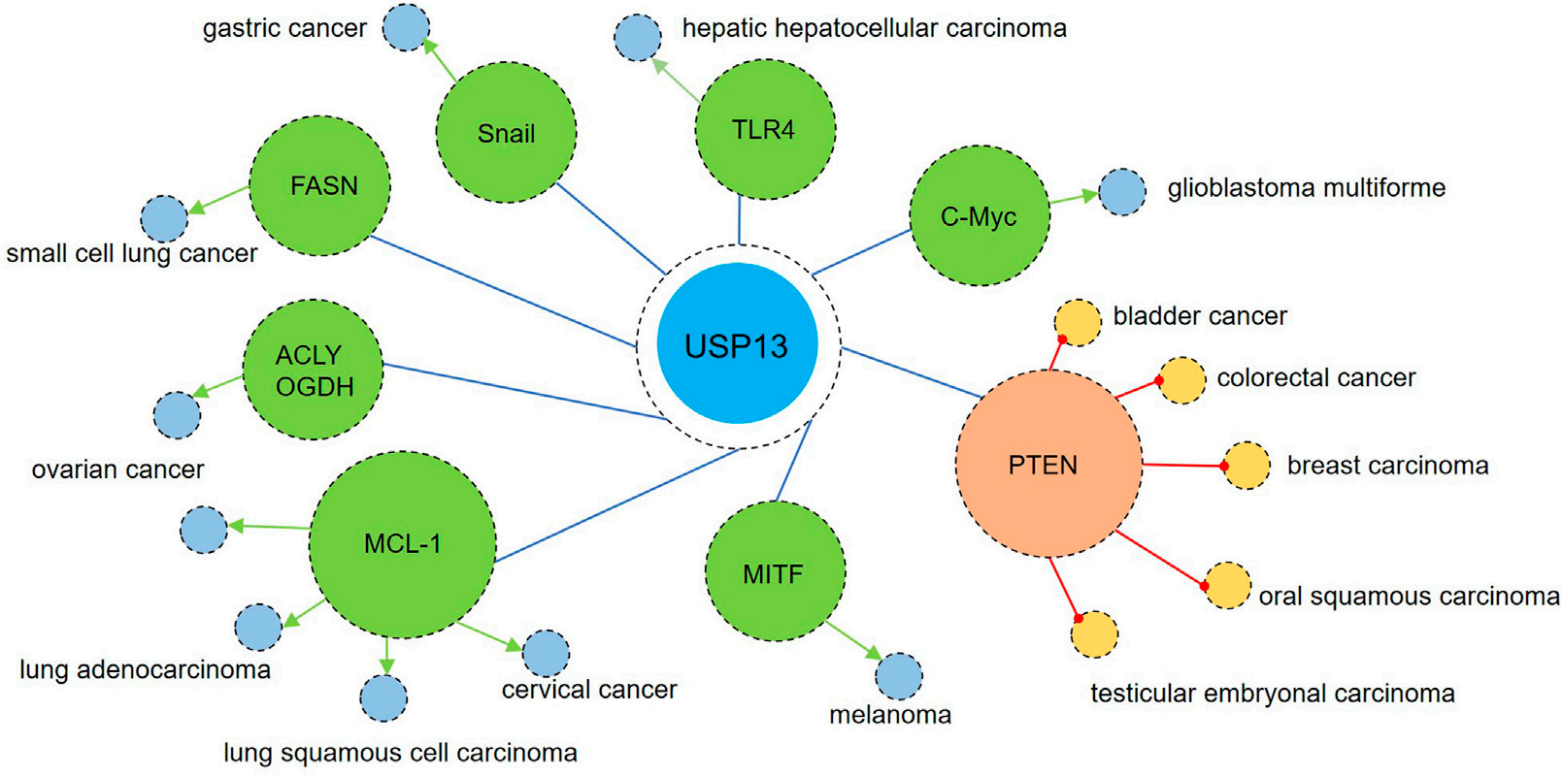
USP13 in cancers. On one hand, USP13 is upregulated in many types of tumors such as lung adenocarcinoma, lung squamous cell carcinoma, small cell lung cancer, ovarian cancer, cervical cancer, hepatic hepatocellular carcinoma, glioblastoma multiforme, melanoma, and gastric cancer. Enhanced USP13 promotes the proliferation, migration, and invasion of tumor cells by upregulating multiple oncogenic factors including MCL-1, c-Myc, TLR4, MITF, and Snail. Besides, USP13 also advanced cancer metabolism *via* modulation of key enzymes ACLY, OGDH, and FASN. On the other hand, downregulation of USP13 is observed in some kinds of cancers including breast carcinomas, bladder cancer, oral squamous carcinoma, colorectal cancer, and testicular embryonal carcinoma. Overexpression of USP13 showed anti-tumor effects *via* upregulation of the tumor suppressor PETN. Blue circle indicates that USP13 can be upregulated in these cancers supported by most studies; Yellow circle represents that USP13 can be downregulated in these cancers suggested by most reports. Green arrow indicates promotion; Red circular arrow represents inhibition.

In summary, due to the diversity of oncogenes and antioncogenes, USP13, serving as a deubiquitinase, may play completely contrary roles in different cancers, which was similar to other members in USP family showing both tumorigenic and tumor-suppressive effects in different contexts, such as USP9X, USP10, USP18, USP22, and USP28 (Cheng et al., 2019; Bonacci and Emanuele, 2020). Figure 6 displays the function of USP13 in different tumors by targeting different substrates. Therefore, more investigations are warranted to broaden the profiles of USP13 in cancers. In addition, further exploring the mechanism by which USP13 is regulated in various tumors and identifying downstream substrates of USP13 may provide new perspectives for future research, and we expect USP13 to be a novel target in the prevention and treatment of cancers.

## Conclusion and future perspectives

In conclusion, USP13 is a deubiquitinase playing different roles in kinds of biological and pathological processes by antagonizing protein ubiquitination. Currently, research regarding USP13 mainly focuses on cancers, which are still obscure and disputed. In general, although there is more evidence supporting the point that USP13 contributes to tumorigenesis, a few studies have verified the inhibition of USP13 in some kinds of tumors, which cannot be neglected. On the other hand, the functions of USP13 in other fields still need further explorations, although some studies have been reported. Given that the complexity and variety of involved proteins, we may not absolutely draw any conclusions about the tangible role of USP13 in the regulation of the cell cycle, DDR, ERAD, autophagy, immunity, inflammation, fibrosis, neurodegeneration, and cancer. Therefore, expanding the substrate spectrum of USP13 could be a part of future research, which is of significance and may enrich the knowledge of USP13 in abovementioned conditions. Besides the deubiquitinating activity, exploring novel functions of USP13 and illustrating its crystal structure would provide a better understanding of USP13, as well as a structural and functional basis for developing agonists or antagonists targeting USP13. Spautin-1, the only nonspecific inhibitor of USP13 at present, though has been proven effective in suppressing tumors including prostate cancer (Liao et al., 2019) and melanomas (Guo et al., 2020), this antitumor effect may be independent of USP13. Developing novel, specific USP13 inhibitors may provide a promising strategy for the treatment of some tumors, as well as other pathologies, such as neurodegenerative diseases. Therefore, analysis of the crystal structure, identification of new substrates, and development of agonists and antagonists may provide new insights for future research.
